# Multi-Omics Study on Molecular Mechanisms of Single-Atom Fe-Doped Two-Dimensional Conjugated Phthalocyanine Framework for Photocatalytic Antibacterial Performance

**DOI:** 10.3390/molecules29071601

**Published:** 2024-04-03

**Authors:** Shihong Diao, Yixin Duan, Mengying Wang, Yuanjiao Feng, Hong Miao, Yongju Zhao

**Affiliations:** 1 Chongqing Key Laboratory of Herbivore Science, College of Animal Science and Technology, Southwest University, Chongqing 400715, China; dsh991009@126.com (S.D.); duanyx315@163.com (Y.D.); wmyklzzy@163.com (M.W.); 2 The Faculty of Pharmacy, Chongqing Medical and Pharmaceutical College, Chongqing 401331, China

**Keywords:** conjugated phthalocyanine framework, photocatalysis, antibacterial mechanism, transcriptome, metabonomic

## Abstract

Currently, photocatalysis of the two-dimensional (2D) conjugated phthalocyanine framework with a single Fe atom (CPF-Fe) has shown efficient photocatalytic activities for the removal of harmful effluents and antibacterial activity. Their photocatalytic mechanisms are dependent on the redox reaction—which is led by the active species generated from the photocatalytic process. Nevertheless, the molecular mechanism of CPF-Fe antimicrobial activity has not been sufficiently explored. In this study, we successfully synthesized CPF-Fe with great broad-spectrum antibacterial properties under visible light and used it as an antibacterial agent. The molecular mechanism of CPF-Fe against *Escherichia coli* and *Salmonella enteritidis* was explored through multi-omics analyses (transcriptomics and metabolomics correlation analyses). The results showed that CPF-Fe not only led to the oxidative stress of bacteria by generating large amounts of h^+^ and ROS but also caused failure in the synthesis of bacterial cell wall components as well as an osmotic pressure imbalance by disrupting glycolysis, oxidative phosphorylation, and TCA cycle pathways. More surprisingly, CPF-Fe could disrupt the metabolism of amino acids and nucleic acids, as well as inhibit their energy metabolism, resulting in the death of bacterial cells. The research further revealed the antibacterial mechanism of CPF-Fe from a molecular perspective, providing a theoretical basis for the application of CPF-Fe photocatalytic antibacterial nanomaterials.

## 1. Introduction

The development of environment-friendly and sustainable antibacterial materials has become even more important due to the resistance of bacteria to antibiotics and the significant threat that this resistance poses to all aspects of public health [1,2]. Fortunately, the application of various nanomaterials—such as carbon compounds, metal-based nanomaterials, and conjugated organic framework—has come to the forefront in developing new strategies for dealing with bacterial infections [3]. Among these strategies, conjugated organic frameworks, including metal organic frameworks (MOFs) and covalent organic frameworks (COFs), have been extensively studied and applied in antibacterial photocatalytic therapy (APCT) as novel alternatives to traditional antibacterial agents [4,5].

Phthalocyanines (PCs) are naturally occurring nitrogen-bridged macrocycles with the ability to constitute compounds with metal ions (M-PCs) and excellent thermal stability. The redox properties and electron transfer capabilities of M-PCs have been explored because multiple chemical transformations can be carried out through the coordination of metal ions with catalytic activity, such as the degradation of photocatalytic pollutants and being an antibacterial agent [6]. Not only has M-PC shown great potential in several fields but its application as a polymer counterpart with an extended conjugated backbone in multiphase catalysts has also attracted widespread attention. Consequently, the hydrolysis and antibacterial properties of PC-containing skeleton materials have also been widely studied, and most of the photocatalytic mechanisms of PC-based frameworks have been based on nanomaterials. In addition, photocatalytic materials irradiated with light at energies greater than their band gap energies can produce electron and hole pairs. Employing controllable light can cause charge carriers to move to the surface of the photocatalytic material and can make these carriers capable of splitting oxygen or generating water as well as generating powerful reactive oxygen species (ROS) such as singlet oxygen (^1^O_2_) and hydroxyl radicals (•OH), all leading to the damage of bacteria [7]. For improving photocatalytic activity, photocatalytic mechanisms based on the critical factors of light absorption [8], photogenerated electron hole separation [9], and interfacial reactions [10] in photocatalysis have been reported on before. Nevertheless, a comprehensive analysis of the molecular antibacterial mechanism of photocatalytic materials remains to be investigated. Omics technologies, including transcriptomics and metabolomics, are widely used by researchers because of their high sensitivity and wide range of applications [11]. Based on high-throughput sequencing technology, transcriptomics can provide quantitative information about differentially expressed genes. However, they cannot reveal the metabolic changes induced after transcription [12]. On the other hand, metabolomics can quantify all small molecule metabolites in an organism [13]. Therefore, the combination of transcriptomics and metabolomics technologies will enable analysis of the molecular regulatory mechanisms of the photocatalytic treatment of bacteria in a more complete way.

In the present work, CPF-Fe with broad-spectrum antibacterial efficacy under visible light irradiation was successfully synthesized and used as an antibacterial agent. The antibacterial activity was superior to previous work [14]. In this study, *E. coli* and *S. enteritidis* were used as experimental subjects. Subsequently, the photocatalytic antibacterial mechanisms were further studied from two different aspects. For the chemical mechanisms, the single-atom Fe-supported CPF significantly promoted the separation of photoelectrons, whereas its antibacterial efficiency was further improved. Meanwhile, a combined analysis of transcriptomics and metabolomics was used to investigate the specific antibacterial molecular mechanism of CPF-Fe; this nanomaterial was able to enter the cell after destroying the cell membrane structure and produce active species (h^+^ and ROS) and cause oxidative stress damage, which in turn destroyed the bacterial oxidative stress defense system. Moreover, the research suggested that CPF-Fe performed its antibacterial activity through disrupting the composition of the bacterial cell wall, the osmotic pressure of the cell, energy metabolism, and the metabolism of nucleic acids—especially DNA replication. Overall, single-atom catalysis was used to improve the antibacterial activity of CPF-Fe and to further analyze the molecular mechanism.

## 2. Results and Discussion

### 2.1. Characterization of CPF-Fe and Antibacterial Activity Tests

**Figure 1:**
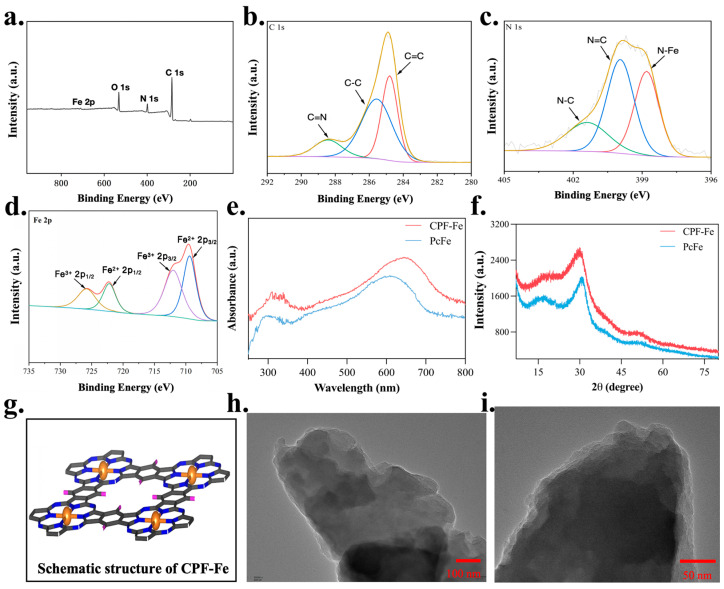
Structure characterization of CPF-Fe. (**a**) The spectrum of CPF-Fe; (**b**) XPS spectra of C 1s; (**c**) XPS spectra of N 1s; (**d**) XPS spectra of Fe 2p; (**e**) UV–visible diffuse reflectance spectrum of CPF-Fe and PcFe; (**f**) XRD spectra of CPF-Fe and PcFe; (**g**) the schematic structure of CPF-Fe; and (**h**,**i**) TEM images of the CPF-Fe nanosheet.

CPF-Fe with single metallic sites was successfully synthesized using modified solvothermal methods, as reported in a previous work [15]. Typically, DBU was used as the monomer while TCNB was used as the catalyst. Ferric chloride (structure-directing agent) was added during the synthesis to help the construction of the conjugated phthalocyanine framework, using the VASP software (Vienna Ab-initio Simulation Package 5.4.4) package to map the crystal structure of CPF-Fe (Figure 1g). Meanwhile, the overall and local chemical environment of CPF-Fe was studied using XPS (Figure 1a). The high-resolution XPS spectra of C (Figure 1b), N (Figure 1c), and Fe (Figure 1d) indicated the formation of macrocyclic structure of phthalocyanine, which were just as expected. The three characteristic diffraction peaks in the spectrum of C 1s represented C=N, C-C, and C=C. The fine spectrum of N 1s, with three peaks, confirmed the pyridine/pyrrole-type nitrogen and N-Fe bonding. The fitting curve in the Fe 2p spectrum was deconvoluted into four peaks corresponding to the typical Fe^2+^ 2p_3/2_ (709.66 eV), Fe^3+^ 2p_3/2_ (711.90 eV), Fe^2+^ 2p_1/2_ (722.82 eV), and Fe^3+^ 2p_1/2_ (724.74 eV) peaks, which indicated the Fe-N_4_ structure in the CPF-Fe nanosheet [16]. The formation of active centers with effective charge transfer capability was facilitated by N-Fe metal centers and conjugate linkages. Moreover, the light absorption and crystallinity of CPF-Fe or PcFe were analyzed using UV–vis diffuse reflectance (Figure 1e) and powder X-ray diffraction (XRD) (Figure 1f), respectively. The light absorption of CPF-Fe was broader than that of PcFe, and there were no obvious diffraction peaks in the X-ray diffraction spectra of CPF-Fe, indicating that CPF-Fe was an amorphous material. Furthermore, CPF-Fe exhibited a nanosheet morphology, as observed using transmission electron microscopy (TEM) (Figure 1h,i), which is beneficial to providing a larger number of contact sites than those in PcFe.

**Figure 2:**
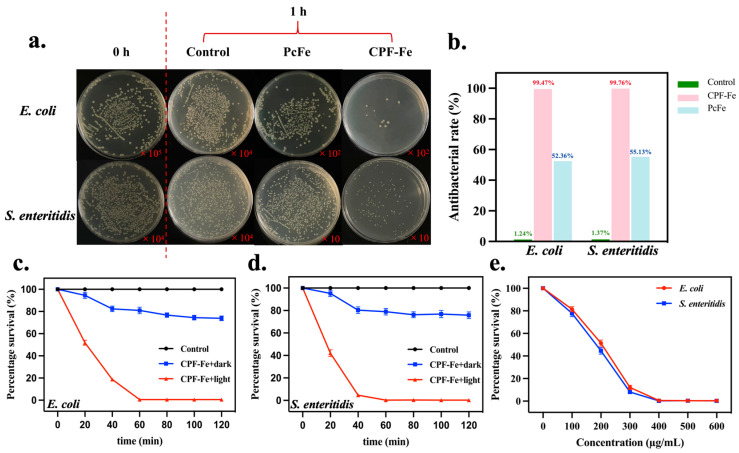
CPF-Fe high antibacterial activity. (**a**) Images of the bacterial colonies formed by *E. coli* and *S. enteritidis* with CPF-Fe and PcFe. (**b**) The antibacterial rates of different treatments against *E. coli* and *S. enteritidis*. (**c**) The antibacterial degradation of *E. coli.* (**d**) The antibacterial degradation of *S. enteritidis*. (**e**) The percentage survival of *E. coli* and *S. enteritidis* under the treatment of CPF-Fe with different concentrations (µg/mL).

As expected, the CPF-Fe nanosheet provided excellent photocatalytic bactericidal activity against *E. coli* and *S. enteritidis* under the irradiation of visible light (Figure S1). The bacterial densities of *E. coli* (Figure 2c) and *S. enteritidis* (Figure 2d) treated with CPF-Fe under visible light irradiation were much lower than those under dark conditions or in the control group, indicating the bactericidal activity of the CPF-Fe photocatalytic properties under light irradiation (Figure 2a). The antibacterial rates of CPF-Fe against *E. coli* and *S. enteritidis* at the concentration of 500 µg/mL for 1 h were 99.47% and 99.76%, respectively (Figure 2b). The antibacterial effect intensified with the increase in the concentration of CPF-Fe (Figure 2e and Figure S2). More importantly, the photocatalytic antibacterial activity of CPF-Fe was greater than that of PcFe, for which the antibacterial rates of PcFe against *E. coli* and *S. enteritidis* were 52.36% and 55.13%, respectively. These results clearly supported the conclusion that the conjugated phthalocyanine framework structure with a single Fe atom could enhance the antibacterial efficiency of CPF-Fe upon visible light irradiation.

### 2.2. CPF-Fe Treatment of Farm Sewage

To further test the effect of the CPF-Fe photocatalytic antibacterial agent on microbial indicators and chemical oxygen demand (CODcr) indicators in wastewater treatment, CPF-Fe dispersion with a concentration of 500 µg/mL was added to the sewage samples and irradiated with visible light for 1 h. We found that the total numbers of bacteria and *E. coli* in the samples of the CPF-Fe-treated group were significantly reduced and that the total antibacterial rate was 80% (Table 1). In addition, the CODcr of the control group sample was 1.39 × 10^4^ mg/L. After CPF-Fe treatment, the CODcr indicator decreased to 2.44 × 10^3^ mg/L, and the removal rate was 82.45%.

### 2.3. Antibacterial Molecular Mechanisms of CPF-Fe

In order to further explore the antibacterial molecular mechanisms of CPF-Fe, a combined analysis of multi-omics technology (transcriptomics and metabolomics) was used to investigate the effect of CPF-Fe on the *E. coli* and *S. enteritidis*. 

**Figure 3:**
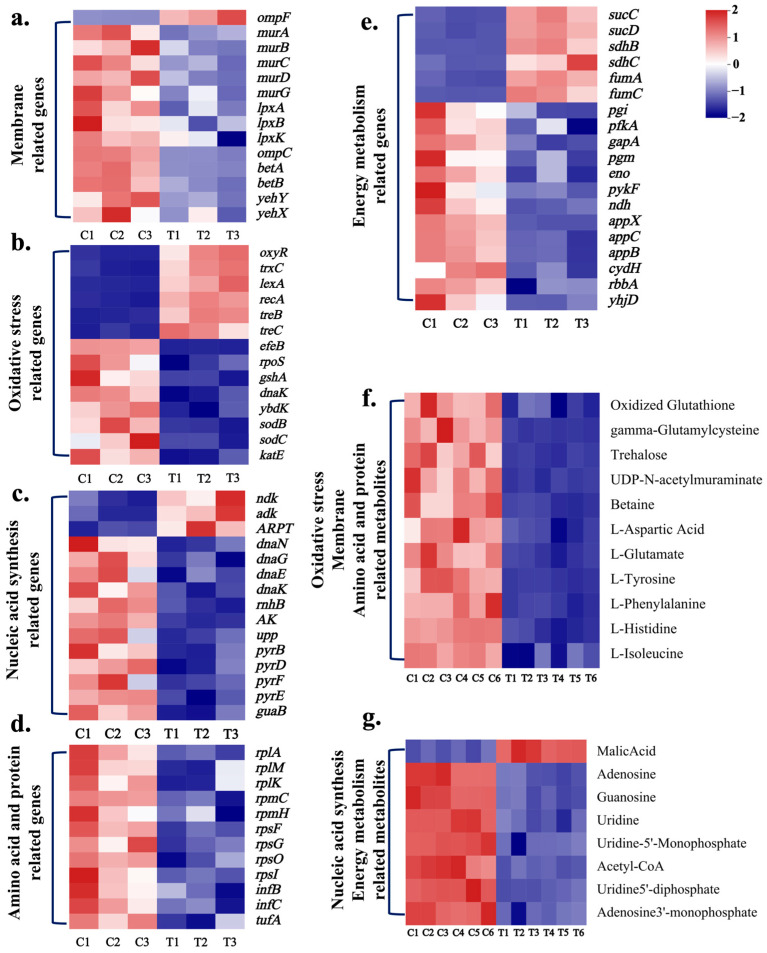
Antibacterial molecular mechanisms of CPF-Fe. (**a**–**e**) Differential expression genes related to each pathway in *E. coli* after CPF-Fe treatment. (**f**,**g**) Differential metabolites related to each pathway in *E. coli* after CPF-Fe treatment.

The changes in the transcriptome levels of *E. coli* and *S. enteritidis* treated with CPF-Fe were investigated using the Illumina Hiseq platform. Volcano plots showed significant differences in the transcript levels between the control and the CPF-Fe-treated groups for both bacteria (Figure S3a,b). Compared with the control group, after CPF-Fe treatment, a total of 1143 clearly differentially regulated genes (589 up-regulated and 554 down-regulated) were found in *E. coli* and 850 (358 up-regulated and 492 down-regulated) in *S. enteritidis* (Figure 3a–e, Table S1). 

The results of GO analysis showed that the DEGs of *E. coli* and *S. enteritidis* CPF-Fe groups had similar functions, mainly distributed in cellular components (CCs) and molecular functions (MFs) such as protein metabolism and cell membrane components, with a small portion related to biological function (BF) (Figure S4a,b). KEGG enrichment results showed that, after treatment with CPF-Fe, both bacteria produced DEGs associated with the metabolism of amino acids, the TCA cycle, glycolysis, and purine–pyrimidine metabolism (Figure S5a,b). In addition, DEGs induced by CPF-Fe in *E. coli* were involved in oxidative phosphorylation. The combination of GO and KEGG results revealed that the pathways by which CPF-Fe affected both of the gram-negative bacteria were almost the same.

Another method, LC–MS, was used to analyze the changes in the intracellular small molecular weight metabolites of *E. coli* and *S. enteritidis* treated by CPF-Fe. The OPLS-DA results showed that each group of replicated samples was well aggregated and that there was significant inter-group separation (Figure S6a–d). In the *E. coli* samples, a total of 137 differential metabolites were screened, of which 41 increased and 96 decreased (Figure 3f,g and Figure S3c). The number of differential metabolites in *S. enteritidis* samples was 189, including 47 up-regulated and 141 down-regulated metabolites (Figure S3d). The KEGG enrichment scatter plots showed that *E. coli* and *S. enteritidis* annotated to partially identical pathways of DM, including 15 different pathways—such as oxidative phosphorylation, TCA, nucleotide metabolism, and multiple amino acid metabolism, among others (Figure S7). Through transcriptomics and metabolomics analyses, the photocatalytic antibacterial mechanisms of CPF-Fe were clearly illustrated at the molecular aspect.

#### 2.3.1. CPF-Fe Destroyed the Cell Membrane/Wall

**Figure 4:**
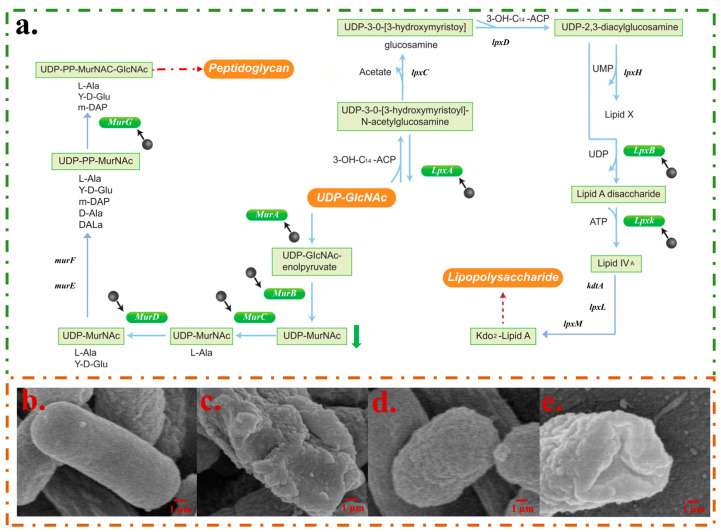
CPF-Fe destroyed the cell membrane/wall. (**a**) CPF-Fe blocked the synthesis of peptidoglycan and lipopolysaccharide in *E. coli*. (**b**,**c**) SEM characterization, respectively, on *E. coli* cells without and with CPF-Fe treatment. (**d**,**e**) SEM characterization, respectively, on *S. enteritidis* cells without and with CPF-Fe treatment.

The morphologies of bacteria before and after the CPF-Fe treatment showed that CPF-Fe was able to disrupt the synthesis of peptidoglycans and lipopolysaccharides in the cell wall components of bacteria and altered the osmotic pressure of the cells. The research results showed that eight genes related to cell wall composition synthesis (*murA*, *murB*, *murC*, *murD*, *murG*, *lpxA*, *lpxB*, *lpxK*) were down-regulated in the *E. coli* treatment group (Figure 3a). Among them, *murA*, *murB*, *murC*, *murD*, and *murG* gene expressions were significantly down-regulated, resulting in reduced UDP-MurNac content and hindering the synthesis of peptidoglycan (Figure 3a and Figure 4a) [17]. Another important component of cell walls in gram-negative bacteria is lipopolysaccharide. The *lpxA*, *lpxB*, and *lpxK* genes, which are related to its synthesis process, were down-regulated by 2.05 fold, 2.13 fold, and 2.07 fold, respectively, thus directly affecting the synthesis of lipopolysaccharide (Figure 4a) [18]. The above results indicated that CPF-Fe could inhibit the synthesis of gram-negative bacterial cell walls.

Moreover, it was found that CPF-Fe down-regulated the levels of genes and metabolites that are related to the maintenance of cell osmotic pressure in *E. coli*, which may lead to a decrease in intracellular osmotic pressure and bacterial shrinkage. Related studies have shown that, in a low osmotic pressure environment, the expression of *ompF* gene increases, while that of *ompC* decreases [19]. The results of this experiment were consistent, indicating that the bacteria were in a low osmotic pressure state after CPF-Fe treatment (Figure 3a). Betaine is an important alkaloid that maintains the cell osmotic pressure in *E. coli*. However, its content significantly decreased in the treatment group. This may be due to the down-regulation of important genes *betA* and *betB* related to its biosynthesis by 2.56 fold and 2.16 fold, respectively [20,21]. Furthermore, significant down-regulation of *yehY* and *yehX* genes, which encode the uptake system of osmoprotectant molecules, may impede cellular uptake of osmotic agents, leading to a decrease in intracellular osmotic pressure [22]. This was corroborated by significantly lower levels of amino acid metabolites such as L-aspartic acid, L-glutamate, L-tyrosine, and L-phenylalanine, all of which were significantly down-regulated as osmotic regulators (Figure 3f). The changes in differentially expressed genes and metabolites could lead to lower than normal intracellular osmotic pressure, forcing cells to shrink, destroying subcellular structures, and ultimately leading to the death of cell [23]. The SEM results confirmed the conclusion that, in the *E. coli* control group, the bacteria had a typical rod-like structure with a smooth bacterial surface, while in the treatment group, the bacterial surface was wrinkled (Figure 4b–e).

In *S. enteritidis*, except for reducing betaine levels, other regulatory pathways were found to be consistent with those of *E. coli* (Tables S1 and S2). Moreover, the content of dTDP-L-rhamnose, the raw material for biosynthesis of O-antigen and one of the main components of lipopolysaccharide, was significantly lower in the *S. enteritidis* treatment group, which was likely due to the down-regulation of the expression of *ansB* and *ptuA* genes (Table S1) [24]. Based on this analysis, it was speculated that CPF-Fe could inhibit the synthesis of bacterial cell walls and reduce the bacterial osmotic pressure.

#### 2.3.2. CPF-Fe Resulted in the Oxidative Stress

**Figure 5:**
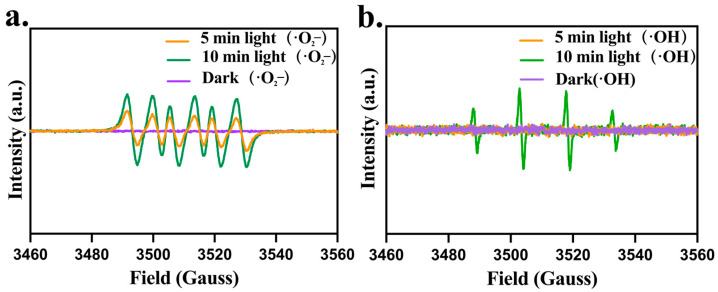
ROS detection. (**a**) •O_2_^−^ EPR spectra of CPF-Fe under 5 and 10 min solar simulator irradiation. (**b**) •OH EPR spectra of CPF-Fe under 10 min solar simulator irradiation.

Due to CPF-Fe containing a large number of active sites and excellent photocatalytic performance, CPF-Fe was able to generate ROS when exposed to solar irradiation. In addition, previous study has shown that different photocatalytic antibacterial agents have different effective reactive oxygen species [25]. In this research, under visible light irradiation, the peak intensities of DMPO -•O_2_^−^ and DMPO -•OH signals significantly increased, indicating that ROS was the main active substance produced during the CPF-Fe-based photocatalytic oxidation reaction (Figure 5a,b). Electrons reacted with O_2_ adsorbed on the surface of photocatalysts to generate superoxide radical anions (•O_2_^−^) (Equation (1)). Meanwhile, h+ reacted with H_2_O adsorbed on the surface of the photocatalyst to generate hydroxyl radicals (•OH) (Equation (2))

O_2_ + e^−^ → •O_2_^−^ (1)

h^+^ + H_2_O → •OH (2)

The production of ROS may disrupt enzymes (superoxide dismutases (SODs), peroxidases (PODs)) and non-enzyme systems or substances in *E. coli*, including the glutathione system (GSH) and trehalose. We found 13 differentially expressed genes associated with oxidative stress in *E. coli* after CPF-Fe treatment (*oxyR*, *trxC*, *dnaK*, *rpoS*, *lexA*, *recA*, *efeB, sodB*, *sodC*, *gshA*, *ybdK*, *treB*, *treC*) (Figure 5b). The most important one was the up-regulation of *oxyR* gene expression, indicating that CPF-Fe may lead to oxidative stress in *E. coli* [26]. The up-regulation of the *trxC* gene also validated that the *oxyR* gene regulated *trxC* gene expression and bound to it in response to oxidative stress [27]. Meanwhile, the expression of the *dnaK* and *rpoS* genes related to the resistance to oxidative stress was inhibited, which could cause damage to cells [28].

Also, the *recA* gene and the *lexA* gene in bacteria, which are related to the repairing of DNA, were up-regulated by 3.47 fold and 3.79 fold, respectively (Figure 3b), indicating that oxidative stress may cause damage to *E. coli* DNA, thereby activating the SOS regulatory system for DNA repair [29,30].

Moreover, the *efeB*, *sodB*, and *sodC* genes, which encode enzymes that regulate the degradation of ROS, were down-regulated by 2.41 fold, 2.57 fold, and 2.92 fold, respectively (Figure 3b). Among them, the *efeB* gene encodes PODs and the *sodB* and *sodC* genes regulate SODs—both of which have the function of reducing ROS-induced oxidative damage to the bacteria [31,32]. Therefore, it can be inferred that CPF-Fe may cause the accumulation of ROS in *E. coli*, resulting in the death of cells.

Moreover, non-enzymatic systems associated with defense against oxidative stress were also affected by CPF-Fe treatment. Two genes related to the GSH antioxidant system (*gshA* and *ybdK*) were down-regulated by 2.56 fold and 2.18 fold in *E. coli*, respectively, resulting in a significant decrease in oxidized glutathione and γ-glutamylcysteine (Figure 3b,f) [33]. Moreover, the genes *treC* and *treB*, which encode trehalase, were up-regulated by 4.77 fold and 4.75 fold, respectively, resulting in a down-regulation of trehalose levels in *E. coli* (Figure 3b,f) [34]. The degradation of *E. coli* by trehalose weakened the cleaning of ROS, leading to the excessive accumulation of ROS [35]. These results suggested that CPF-Fe not only disrupted the GSH antioxidant system but also caused the accumulation of ROS. In *S. enteritidis* treated with CPF-Fe, there were similar regulatory pathways as in *E. coli* in addition to the regulatory effect on trehalose (Tables S1 and S2). Moreover, the significant up-regulation of the *yggX* gene in *S. enteritidis* further confirmed the oxidative stress of *S. enteritidis*, while the down-regulation of the *yfcG* gene further affected the GSH system [36]. Therefore, CPF-Fe is able to produce ROS under visible light irradiation, causing oxidative stress in gram-negative bacteria and destroying their oxidative stress defense system, which ultimately leads to the death of bacteria.

#### 2.3.3. CPF-Fe Disrupted Synthesis of Nucleic Acid 

**Figure 6:**
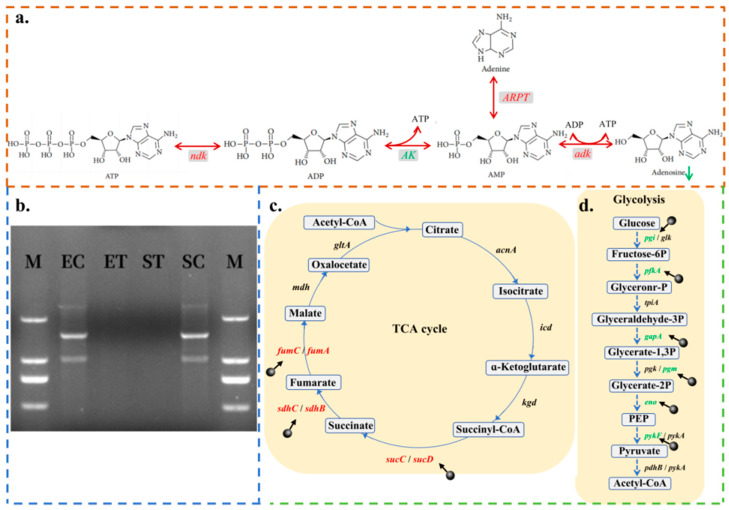
Nucleic acid synthesis and energy metabolism. (**a**) CPF-Fe inhibited adenosine biosynthesis in *E. coli*. (**b**) Agarose gel electrophoresis of 16s rDNA extracted from *E. coli* and *S. enteritidis* without (Lane EC, SC) and with (Lane ET, ST) treatment of CPF-Fe; M: marker. (**c**) CPF-Fe disturbed the TCA cycle in *E. coli*. (**d**) CPF-Fe inhibited glycolysis in *E. coli*.

CPF-Fe may bind to nucleic acid and disrupt the synthesis of nucleic acids in bacteria, leading to bacterial death. Sixteen differentially expressed genes (*dnaN*, *dnaG*, *dnaE*, *dnaK*, *rnhB*, *ndk*, *adk*, *APRT*, *AK*, *upp*, *pyrB*, *pyrD*, *pyrF*, *pyrE*, *guaB*, *yjjG*) were found after treatment with CPF-Fe (Figure 3c). Among them, the *dnaN, dnaG*, *dnaE*, *dnaK* and *rnhB* genes involved in initiating the replication of DNA were significantly down-regulated, indicating that CPF-Fe may interfere with the bacterial DNA semi-reserved replication process [37,38]. PCR agar gel electrophoresis showed that there were no clear DNA bands after treatment with CPF-Fe, whereas the control group did have intact DNA bands, further demonstrating that CPF-Fe may disrupt the process of the replication of DNA (Figure 6b).

On the other hand, the metabolisms of pyrimidine and purine were also influenced by CPF-Fe. Adenosine, guanosine, and uridine are the basic structural units that make up nucleic acids. In *E. coli*, the *ndk*, *adk*, and *APRT* genes, which negatively regulate the biosynthesis of adenosine, were up-regulated by 2.31 fold, 2.07 fold, and 2.25 fold, respectively, while the *AK* gene, which has a positive regulatory role, was down-regulated by 2.50 fold (Figure 6a and Table S1) [39]. As a result, the level of adenosine was down-regulated in the *E. coli* treatment group (Table S2). The *upp* and *guaB* genes involved in the biosynthesis of guanosine and uridine were also down-regulated by 2.49 fold and 2.56 fold, respectively [40,41]. Among them, the biosynthesis of uridine 5′-monophosphate (UMP), a common precursor of other pyrimidine nucleotides, is mainly catalyzed by various enzymes for the synthesis of UMP from carbamoyl-aspartic acid. However, the *pyrB*, *pyrD*, *pyrF*, and *pyrE* genes encoding these enzymes were expressed at a low level, which caused a decrease in the content of UMP (Figure 3g) [42]. We also found that CPF-Fe may interfere with some metabolic reactions such as a significant decrease in the levels of adenosine 3′-monophosphate, uridine 5′-diphosphate, and adenosine in the metabolic reactions of R00183 and R00157 (Figure 3g). Therefore, this indicated that CPF-Fe interfered with pyrimidine and purine metabolism, thereby disrupting nucleic acid synthesis.

After CPF-Fe treatment, similar performances were also observed in *S. enteritidis* (Tables S1 and S2). In addition, the *yjjG* gene, which helps hydrolyze the 5′-nucleotide phosphate group, was down-regulated by 2.36 fold in *S. enteritidis* [43]. In summary, CPF-Fe could disrupt the synthesis of nucleic acid in bacteria, ultimately causing them death.

#### 2.3.4. CPF-Fe Inhibited Amino Acid Metabolism and Protein Synthesis

In addition to being involved in the regulation of cellular osmotic pressure, amino acids are the basic units of protein synthesis in microorganisms, as well as an important carbon and nitrogen source for bacteria [44,45]. During the process of amino acid metabolism, intermediates are constantly produced to provide carbon or nitrogen sources for bacteria, such as the transamination reaction of aspartic acid and α-ketoglutarate to produce oxaloacetic acid and glutamic acid [46,47]. In addition, many amino acids are also precursors for substances involved in energy metabolism, such as glutamic acid—which yields α-ketoglutaric acid by oxidative deamination. Furthermore, glycine and cysteine are decomposed into pyruvic acid [48]. Therefore, a disordered process of amino acid metabolism may inhibit microbial growth. Most of the amino acids detected in this study were significantly down-regulated in both the bacterial treatment groups, except tryptophan, glycine, and valine. The metabolite levels of aspartate, glutamate, histidine, tyrosine, and isoleucine were the most significantly down-regulated, while genes related to their metabolism were also found to be down-regulated (Figure 3g). The results indicated that CPF-Fe may affect the growth of bacteria by inhibiting the metabolism of some amino acids and hindering the production of carbon and nitrogen sources.

Translation, as an important step in protein synthesis, is the formation of proteins by arranging amino acids in the sequence of bases in mRNA in the ribosome [49]. Ribosomes are composed of 30 s and 50 s ribosomal proteins, which have the function of catalyzing and promoting protein synthesis [50]. The results of this study showed that the genes encoding 50 s ribosomal proteins (*rplA*, *rplM*, *rplK*, *rpmC*, *rpmH*) and 30 s ribosomal proteins (*rpsF*, *rpsG*, *rpsO*, *rpsI*) were significantly down-regulated, indicating that the translation capability of gram-negative bacteria was weakened after CPF-Fe treatment (Figure 3d) [51,52]. In addition, in *E. coli*, the expression of the *infB* and *infC* genes, which encode the translation initiation factors IF-2 and IF-3, was down-regulated by 2.52 fold and 2.91 fold, respectively, and the expression of the *tufA* gene, which encodes the translation elongation factor Tu-1, was down-regulated by 2.30 fold, which suggested that CPF-Fe hindered the biosynthesis of proteins [53].

#### 2.3.5. Energy Metabolism Disorder

Maintaining energy balance is essential for the survival of bacteria [54]. In this study, it was found that CPF-Fe may interfere with the energy metabolism of bacteria by affecting three pathways: glycolysis, the TCA cycle, and oxidative phosphorylation. Six differentially expressed genes associated with the glycolytic pathway were detected to be significantly down-regulated in *E. coli* after CPF-Fe treatment (*pgi*, *pfkA*, *gapA*, *pgm*, *eno*, *pykF*), suggesting that CPF-Fe may block the glycolytic pathway (Figure 3e and Figure 6d). The metabolomics results showed that, as the final product of glycolysis, acetyl CoA had a significant decrease in its content, further demonstrating the influence of CPF-Fe on the glycolytic pathway (Figure 3g) [55]. In addition, acetyl CoA enters the TCA cycle through condensation with oxaloacetic acid and then undergoes oxidative decarboxylation reactions to generate CO_2_, H_2_O, and energy [56]. Therefore, it is speculated that CPF-Fe would also have an impact on the TCA cycle. As expected, some of the genes involved in the *E. coli* TCA cycle, including *sucC*, *sucD*, *sdhB*, *sdhC*, *fumA*, and *fumC*, were significantly up-regulated, leading to a significant increase in the levels of their intermediate product—malate—as well (Figure 6c). This phenomenon may lead to the accumulation of NADH, thereby affecting the balance of TCA [57]. Moreover, it has been shown that high levels of NADH not only lead to reduced cell viability but also promote the release of ROS [58].

Subsequently, we found that CPF-Fe significantly down-regulated the expression of the ndh gene encoding NADH: quinone oxidoreductase, and the *appX*, *appC*, *appB*, and *cydH* genes encoding cytochrome bd oxidoreductase, which catalyzes oxidative phosphorylation in *E. coli* (Figure 3e) [59]. Furthermore, previous studies have shown that *rbbA—yhjD* double deletion mutants inhibit the oxidative phosphorylation pathway in bacterial cells [60]. In this experiment, the expression of these two genes in *E. coli* treated with CPF-Fe significantly decreased, indicating that CPF-Fe could hinder the oxidative phosphorylation pathway of energy metabolism. Similarly, CPF-Fe was able to produce similar changes in the expression of the above-listed related genes in relation to *S. enteritidis* (Tables S1 and S2). Only some of the genes (*sucC*, *sdhB*, and *cydH*) did not show significant change (FC > 1.5 & FC < 0.8). Therefore, it can be speculated that CPF-Fe interferes with the energy metabolism of bacteria through the three pathways mentioned previously, leading to the death of the organisms.

**Figure 7:**
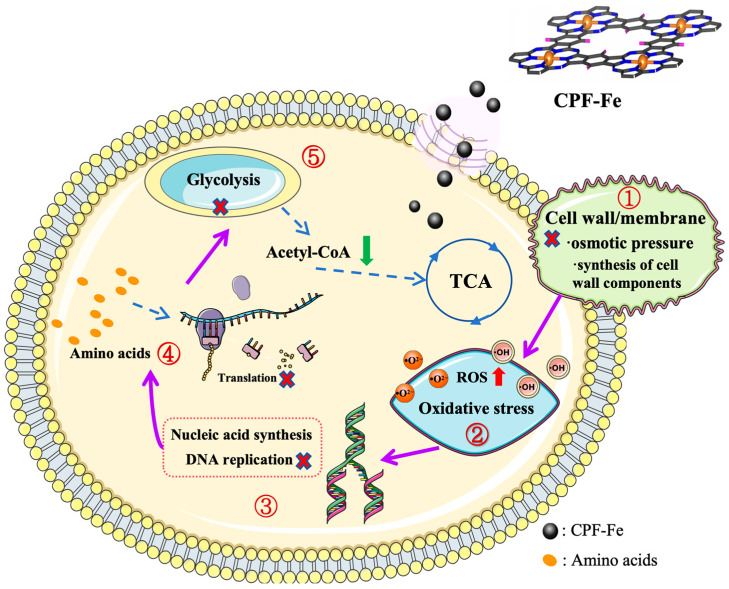
Illustration of the regulatory mechanism of the antimicrobial activity of CPF-Fe.

Above all, multi-omics technology (transcriptome and metabolome association analyses) could provide cross information between differentially expressed genes and metabolites. Combining the gene analysis and the in vivo metabolic set analysis with the in vitro antibacterial activity results, the antibacterial chemical and molecular mechanisms of CPF-Fe were further summarized and identified clearly. Firstly, a CPF-Fe nanosheet with a 2D structure entered the cell after destroying the cell membrane structure and produced active species (h^+^ and ROS), which could disrupt the synthesis of cell wall components and result in oxidative stress in bacteria. Then, single-atom Fe, acting as the electron center in CPF-Fe, affected the charge balance, with nucleic acid and amino acid metabolism being disrupted and protein synthesis being impaired. Furthermore, CPF-Fe blocked various energy metabolism pathways, including TCA cycle, glycolysis, and oxidative phosphorylation (Figure 7).

## 3. Materials and Methods

### 3.1. Materials

Yeast powder, agar powder, tryptone, and glycerol were obtained from Solarbio Technology Co., Ltd., Beijing, China. The *E. coli* ATCC 25312 strain and *S. enteritidis* ATCC 29629 strain were obtained from Biotech Co., Ltd., Beijing, China. All chemicals were of analytical grade. The distilled water and chemicals involved in the study were used under sterile conditions.

### 3.2. Sample Preparation

CPF-Fe was synthesized based on the method reported in previous reports with some modification [14]. First, benzene-1,2,4,5-tetracarbonitrile (TCNB) and 1,8-diazabicyclo (5,4,0) undec-7-ene (DBU) were used as the monomer and catalyst, respectively. TCNB (0.56 mmol), ferric chloride (0.28 mmol), and DBU (0.9 mL) were dissolved in ethylene glycol (20 mL). The mixed solution was reacted under microwave power of 1200 W for 3 min. Then, the liquid was filtered, and the precipitate was washed three times with ultrapure water. The precipitate was dried in a vacuum oven to obtain black bulk CPF-Fe and then ground into powder. Next, bulk CPF-Fe was added to concentrated sulfuric acid (0.25 mL/mg) and ultrasonicated for 10 min. The mixture was stirred in ultrapure water for 5 h. Finally, it was washed 3 times using ultrapure water and then dried in a vacuum oven. The CP-Fe morphologies were characterized by transmission electron microscopy (TEM, FEI Company, Waltham, MA, USA). The UV–vis–NIR spectra CPF-Fe (200 nm–800 nm) were measured by a UV–vis spectrometer (UV-3600, Shimadzu, Tokyo, Japan). Chemical characterizations of CPF-Fe were recorded on an X-ray diffraction (XRD) meter (Bruker D8 Advance, Oberkochen, Germany) and by X-ray Photoelectron Spectroscopy (XPS, Escalab 250Xi, Thermo, Waltham, MA, USA).

### 3.3. Bacterial Culture

First, 30 µL of *E. coli* and *S. enteritidis* were preserved at −80 °C and added to 3 mL of Luria Bertani (LB) liquid culture medium. The system was incubated at a constant temperature of 37 °C for 12 h at a speed of 160 rpm for resuscitation. Then, 20 µL of recovered *E. coli* and *S. enteritidis* were added to 5 mL of LB liquid medium and then shaken under the same conditions for 5 h to reach a logarithmic growth period.

### 3.4. Photocatalysis of Antibacterial Activity Assessments 

The antibacterial effect of as-prepared CPF-Fe was evaluated with *E. coli* and *S. enteritidis* in a photochemical reactor through the dilution plating method (PCX—50 C Discover, Beijing Perfect Light, Beijing, China). For the light source, a 10 W LED lamp with a UV cutoff filter (λ ≥ 420 nm) was used. A quantity of 25 mg of CPF-Fe (0.5 g/L) was added into a 50 mL bacterial suspension (OD600 = 0.5; about 10^8^ cfu/mL). The antimicrobial test concentration of CPF-Fe was within the range of 200–600 µg/mL, and the control was added with 0.01 M phosphate buffer solution (PBS). The mixed liquids to be tested were put in the photocatalytic reactor and reacted under visible light irradiation at 160 rpm for 2 h. The temperature was maintained at 25.0 ± 0.1 °C. Then, 100 µL of the mixture samples were taken at regular intervals, at which point, they were immediately transferred to solid LB medium plates and cultured at 37 °C for 24 h, using the dilution plating method to count bacteria. Each group of experiments was repeated 3 times, and then the average of each group was plotted as a statistical graph using Prism9 software (9.5.1). The calculation of antibacterial rate:

$$Antibacterial\;rate\left(\%\right)=100\%-\frac{CFU\;of\;G_{CPF-Fe}}{CFU\;of\;G_{con}}\times 100\%$$

where *G_CPF-Fe_* denotes the number of colony-forming units (CFUs) in the treatment group and *G_con_* denotes the number of colony-forming units (CFUs) in the control group.

### 3.5. Sewage Treatment

The tested effluent was collected from the fecal discharge of the goat farm of Southwest University in Beibei District, Chongqing, China. Fecal sewage was collected in sterile plastic bottles to a total of 3 L and left to stand for 1 h. The settled solid particles were removed, and the upper layer of liquid was poured into four 500 mL sterile water sample collection bags and stored at 4 °C. Two bags were taken as the test group; 500 µg/mL CPF-Fe antibacterial agent was added; and an equal amount of PBS was added to the other two bags as the control group. All the bags were stirred well and then left to react for 1 h under the condition of natural light irradiation, after which, sampling was carried out for testing and analysis.

### 3.6. Features of Bacterial Morphology

Bacterial suspensions of *E. coli* and *S. enteritidis* with OD600 = 0.5 were treated with CPF-Fe (treatment group) or PBS (control group). The irradiated bacteria with different treatments were washed with sterile water and centrifuged at 8000 rpm for 5 min to remove the excess CPF-Fe. Then, 2.5% glutaraldehyde was injected into a 1.5 mL centrifuge tube that contained the bacteria and fixed at 4 °C for 8 h. After the removal of glutaraldehyde, these fixed bacteria were dehydrated for 10 min in 30–100% ethanol. Later, the morphology of bacterial cells was observed by scanning electron microscopy (SEM). 

### 3.7. Measurement of ROS

To assess the radical species of CPF-Fe in the photocatalytic reaction, the concentration of reactive oxygen species in the samples was measured continuously using the electron paramagnetic resonance (EPR) technique, under visible light irradiation for 0 min, 5 min, and 10 min. Briefly, CPF-Fe was mixed with a trapping agent (5,5-dimethyl pyridine N-oxide (DMPO)) to capture hydroxyl (•OH) and superoxide radicals (•O_2_^−^). 

### 3.8. PCR Amplification and Gel Electrophoresis of 16s rDNA

DNA from *E. coli* and *S. enteritidis* was extracted using the bacterial genomic DNA extraction kit (DP302, TIANGEN, Beijing, China). The obtained portions of DNA were treated with CPF-Fe under visible light irradiation for 60 min. Then, 27F (5′-AGAGTTTGATCCTGGCTCAG-3′) and 1492R (5′-TACGGTTACCTTG TTACGACTT-3′) were used as primers to amplify the bacterial 16s rRNA genes. The resulting 16s rRNA fragments were tested using 1.5% agarose gel electrophoresis. The specific reaction system for PCR is illustrated in the Supplementary Material.

### 3.9. Transcriptomics Research

*E. coli* and *S. enteritidis* were treated with CPF-Fe or PBS under visible light irradiation for 1 h to evaluate the transcriptional changes of bacteria. Samples treated with PBS constituted the control groups. The method to prepare the samples was described in the Supporting Information (SM2). Three replicates were performed for a total of 12 samples in four groups. The cDNA library was constructed using the TruSeqTM Stranded Total RNA Library Prep Kit reagent (Majorbio Biomedical Science and Technology Co., Ltd., Shanghai, China). Then, 2 × 150 bp sequencing was carried out using the Illumina Hiseq sequencing platform. Raw counts were statistically analyzed using DESeq2 software (R4.1.2) based on negative binomial distribution to screen for eligible differentially expressed genes (*p*-value < 0.05 & |log_2_FC| ≥ 1).

### 3.10. Non-Targeted Metabolomics Analysis

The metabolite changes of *E. coli* and *S. enteritidis* were evaluated using liquid chromatography–mass spectrometry (LC–MS). The method for preparing the samples was described in the Supporting Information (SM2). Six replicates were performed for a total of 24 samples in four groups. The samples were sent to Majorbio Biomedical Technologies Co., Ltd., Shanghai, China for analysis. To ensure the reliability and statistical significance of the test results, three quality control samples (QC) were set up within each group. Metabolites were identified using KEGG and HMDB databases. Differential metabolites (VIP > 1 & *p*-value < 0.05 & |log_2_FC| ≥ 1) were screened between the treated and control groups using ropls (R packages). In addition, transcriptomic and metabolomic association analyses, as well as expression correlation analysis and KEGG analysis, were performed to explore the correlation between differentially expressed genes and differential metabolites.

## 4. Conclusions

In this work, CPF-Fe nanosheets were prepared and used as an antimicrobial agent for combating bacteria under visible light. For the chemical mechanisms, CPF-Fe exhibited excellent antibacterial activity due to the large surface area and faster electron transfer. Meanwhile, the antibacterial molecular mechanisms of CPF-Fe against bacteria was further elucidated based on multi-omics analysis. CPF-Fe influenced the expression of 1143 genes and 137 metabolites in *E. coli* and the expression of 850 genes and 189 metabolites in *S. enteritidis.* Hence, CPF-Fe achieved antibacterial effects mainly through breaking down the bacterial cell membrane structure and entering the cell to produce active substances (h^+^ and ROS), which may disrupt the synthesis of cell wall components and lead to bacterial oxidative stress. CPF-Fe also affected bacterial nucleic acid and amino acid metabolism, interfered with translation processes, and forced protein synthesis to be impaired. In addition, CPF-Fe disrupted various energy metabolism pathways in bacteria, including glycolysis, the TCA cycle, and oxidative phosphorylation. This research contributes to the theoretical foundation for the antibacterial mechanisms of CPF-Fe, which lays the foundation for the subsequent investigation of the action targets of CPF-Fe. Meanwhile, combining nanomaterials with the environment of livestock and poultry breeding provides certain technical and theoretical references for the wide application of the CPF-Fe photocatalytic antibacterial agent in the treatment of livestock and animal husbandry sewage.

## Figures and Tables

**Table 1:** Sewage treatment indicators.

	CODcr (mg/L)	Total Bacteria (CFU/100 mL)	*Escherichia coli* (CFU/100 mL)
Control	1387.7 ± 6.11 ^A^	1501.3 ± 18.82 ^A^	160.3 ± 4.16 ^A^
CPF-Fe	244.3 ± 2.50 ^B^	302.7 ± 9.87 ^B^	2.3 ± 0.58 ^B^

Note: Different capital letters indicate highly significant differences (*p* < 0.01).

## Data Availability

Data are contained within the article and Supplementary Materials.

## Supplementary Materials

The following supporting information can be downloaded at: https://www.mdpi.com/article/10.3390/molecules29071601/s1, Figure S1: Antibacterial assessments; Figure S2: Antibacterial assessments; Figure S3: Transcriptomics and metabolomic response; Figure S4: Transcriptomics response; Figure S5: Transcriptomics response; Figure S6: Metabolomics response; Figure S7: Metabolomics response. Table S1: Differentially expressed genes of *E. coli* and *S. enteritidis* treated with CPF-Fe; Table S2: Differential metabolites of *E. coli* and *S. enteritidis* treated with CPF-Fe.
