# Correction: Diao et al. Multi-Omics Study on Molecular Mechanisms of Single-Atom Fe-Doped Two-Dimensional Conjugated Phthalocyanine Framework for Photocatalytic Antibacterial Performance. *Molecules* 2024, *29*, 1601

**DOI:** 10.3390/molecules30132806

**Published:** 2025-06-30

**Authors:** Shihong Diao, Yixin Duan, Mengying Wang, Yuanjiao Feng, Hong Miao, Yongju Zhao

**Affiliations:** 1 Chongqing Key Laboratory of Herbivore Science, College of Animal Science and Technology, Southwest University, Chongqing 400715, China; dsh991009@126.com (S.D.); duanyx315@163.com (Y.D.); wmyklzzy@163.com (M.W.); 2 The Faculty of Pharmacy, Chongqing Medical and Pharmaceutical College, Chongqing 401331, China

## References Correction

There were citation positioning errors in the original publication [1]. Ref. [47] should be revised to Ref. [45]. 

The new order for references should be as follows:

45. O’Connell, T.C. ‘Trophic’ and ‘source’ amino acids in trophic estimation: A likely metabolic explanation. *Oecologia* **2017**, *184*, 317–326.

46. Lamolle, G.; Simon, D.; Iriarte, A.; Musto, H. Main Factors Shaping Amino Acid Usage Across Evolution. *J. Mol. Evol*. **2023**, *91*, 382–390.

47. Olson, M.E. Sialic Acid Catabolism in *Staphylococcus aureus*. *J. Bacteriol*. **2013**, *195*, 1779–1788.

## Text Correction

To express the ideas better and to make them grammatically correct, the 1st–4th sentences in the first paragraph of Section 2.3.4 should be changed to the following:

In addition to being involved in the regulation of cellular osmotic pressure, amino acids are the basic units of protein synthesis in microorganisms, as well as an important carbon and nitrogen source for bacteria [44,45]. During the process of amino acid metabolism, intermediates are constantly produced to provide carbon or nitrogen sources for bacteria, such as the transamination reaction of aspartic acid and α-ketoglutarate to produce oxaloacetic acid and glutamic acid [46,47]. In addition, many amino acids are also precursors for substances involved in energy metabolism, such as glutamic acid—which yields α-ketoglutaric acid by oxidative deamination. Furthermore, glycine and cysteine are decomposed into pyruvic acid [48]. 

With this correction, the order of some references has been adjusted accordingly. 

The authors state that the scientific conclusions are unaffected. This correction was approved by the Academic Editor. The original publication has also been updated.

## References

[1] Diao S., Duan Y., Wang M., Feng Y., Miao H., Zhao Y. (2024). Multi-Omics Study on Molecular Mechanisms of Single-Atom Fe-Doped Two-Dimensional Conjugated Phthalocyanine Framework for Photocatalytic Antibacterial Performance. Molecules.

