# Supplementary material for: Multi-Omics Study on Molecular Mechanisms of Single-Atom Fe-Doped Two-Dimensional Conjugated Phthalocyanine Framework for Photocatalytic Antibacterial Performance
Source: Molecules. 2024 Apr 3;29(7):1601. doi: 10.3390/molecules29071601 (PMC11013413; doi:10.3390/molecules29071601)
Supplement: Supplementary file 1 [file molecules-29-01601-s001.zip › Supplementary Material 1 .pdf]

Supplementary material

Multi-omics Study on Molecular Mechanisms of Single-Atom Fe-Doped Two-Dimensional Conjugated Phthalocyanine Framework for Photocatalytic Antibacterial Performance

Shihong Diao[1], Yixin Duan[1], Mengying Wang[1], Yuanjiao Feng[2]*, Hong Miao[1]*, Yongju Zhao[1]*

[1]College of Animal Science and Technology, Southwest University; Chongqing Key Laboratory of Herbivore Science, Chongqing 400715, China

[2]Chongqing Medical and Pharmaceutical College, Chongqing 401331, China

*Correspondence authors: E-mail addresses: fengyuanj88@126.com (Y. Feng), miaoh333@swu.edu.cn (H. Miao), zyongju@163.com (Y. Zhao).

1. Materials and methods

1.1. The specific reaction system for PCR as follow:

1 μL 27F upstream primer, 1 μL 1492R downstream primer, 2 μL DNA template, 8 μL 2 × TaqPCRMix, and 8 μL ddH$_2$O were added in sequence. The PCR reaction conditions were set as follows: denaturation at 95°C for 3 min, denaturation at 95°C for 30 sec, annealing at 56°C for 45 sec, extension at 72°C for 2 min, cycling for 40 cycles, and extension at 72°C for 10 min at the end of the last cycle.

1.2. The prepared method of transcriptomics samples as follow:

The samples were centrifuged at 8000 rpm for 5 min to obtain the bacterial precipitate, cleaned them three times with PBS, and finally the samples were snap frozen in liquid nitrogen.

2. Figure S1 to S7

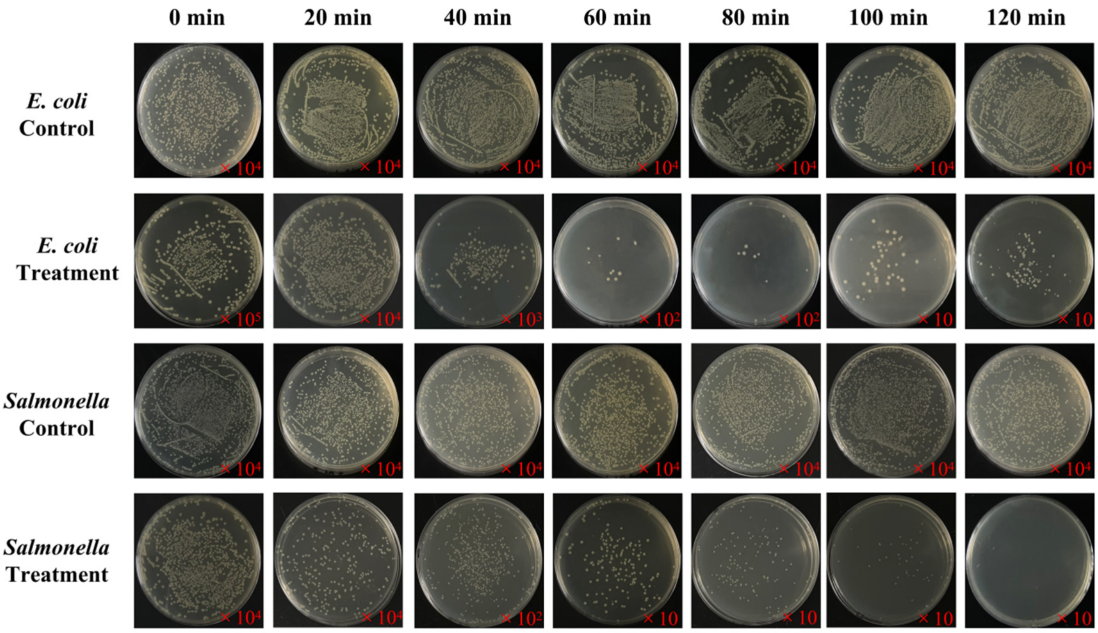

Figure S1 Antibacterial assessments. Images of the bacterial colonies formed by *E. coli* and *S. enteritidis* with different treatment times of CPF-Fe.

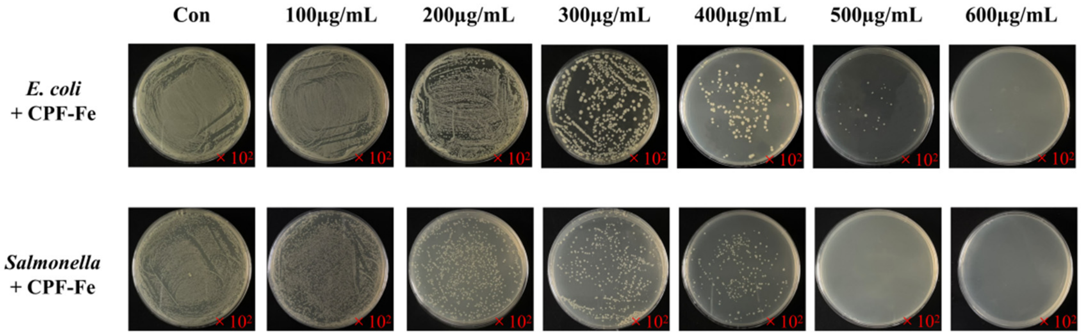

Figure S2 Antibacterial assessments. Images of the bacterial colonies formed by *E. coli* and *S. enteritidis* with different concentrations of CPF-Fe.

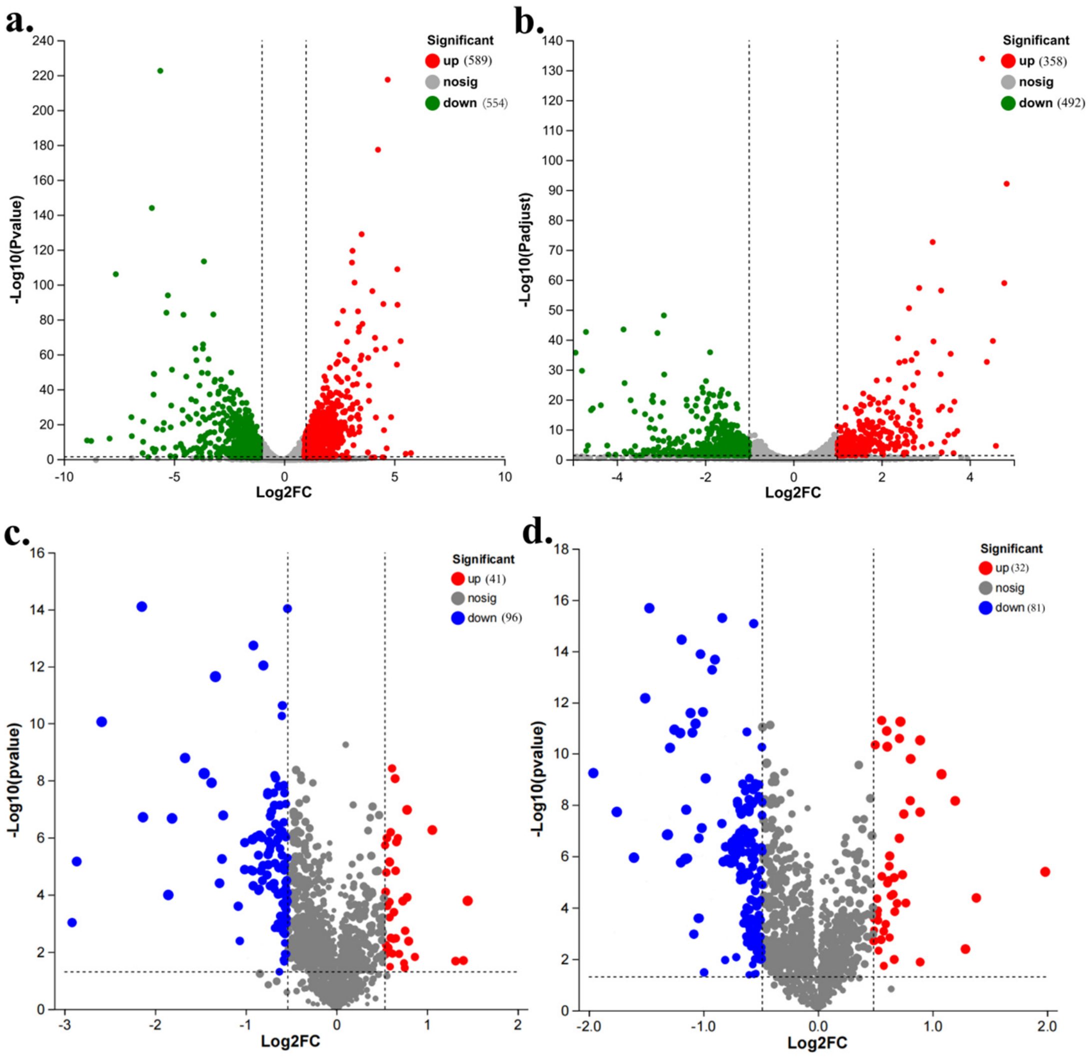

Figure S3 Transcriptomics and metabolomic response. The volcano plot of differentially expressed genes in *E. coli* (a) and *S. enteritidis* (b). The volcano plot of differential metabolites in *E. coli* (c) and *S. enteritidis* (d).

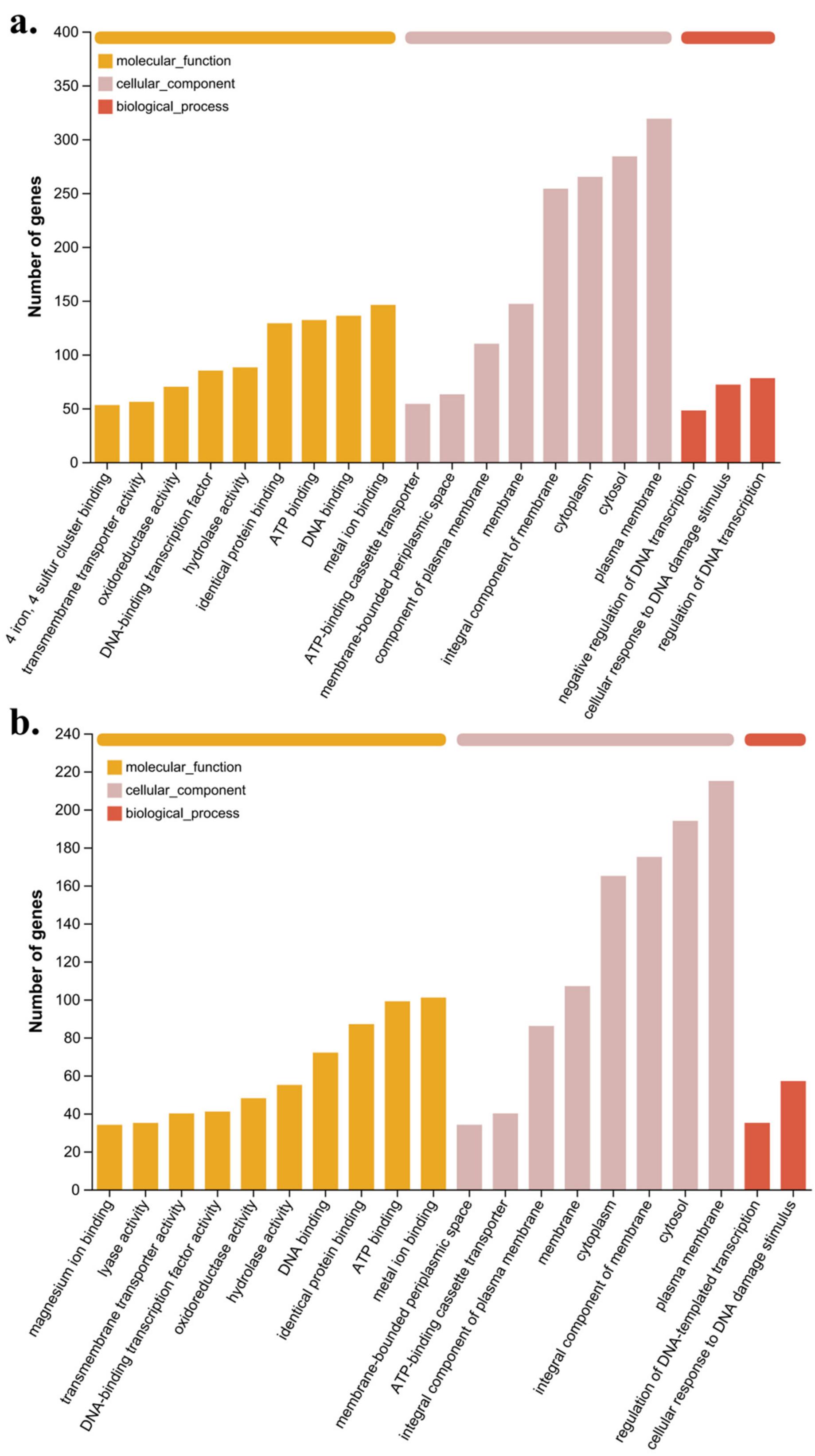

Figure S4 Transcriptomics response. **(a)** GO annotation maps of differentially expressed genes in *E. coli* after CPF-Fe treatment. **(b)** GO annotation maps of differentially expressed genes in *S. enteritidis* after CPF-Fe treatment.

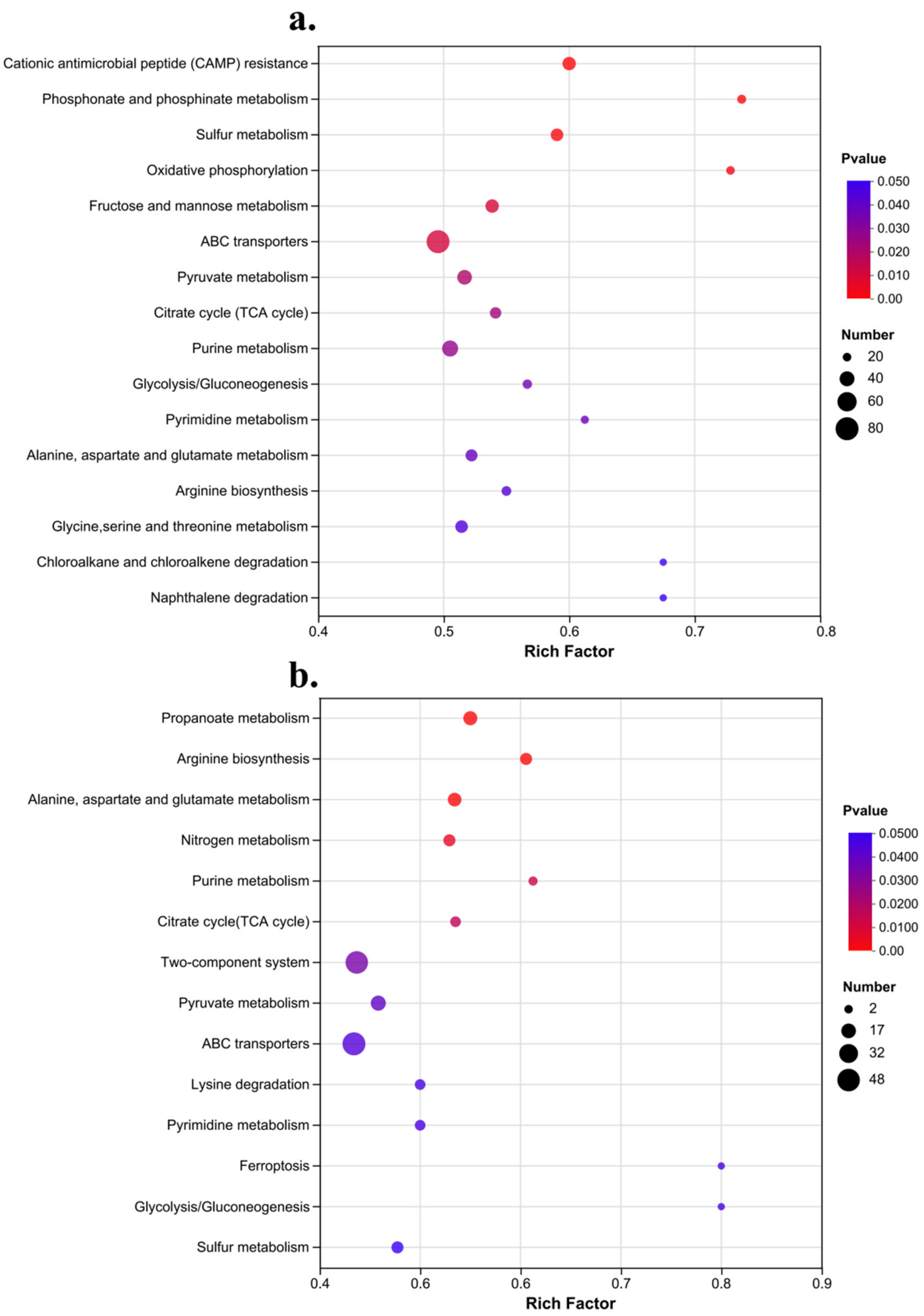

Figure S5 Transcriptomics response. **(a)** KEGG-enriched scatter plot of differentially expressed genes in *E. coli* after treatment with CPF-Fe. **(b)** KEGG-enriched scatter plot of differentially expressed genes in *S. enteritidis* after treatment with CPF-Fe.

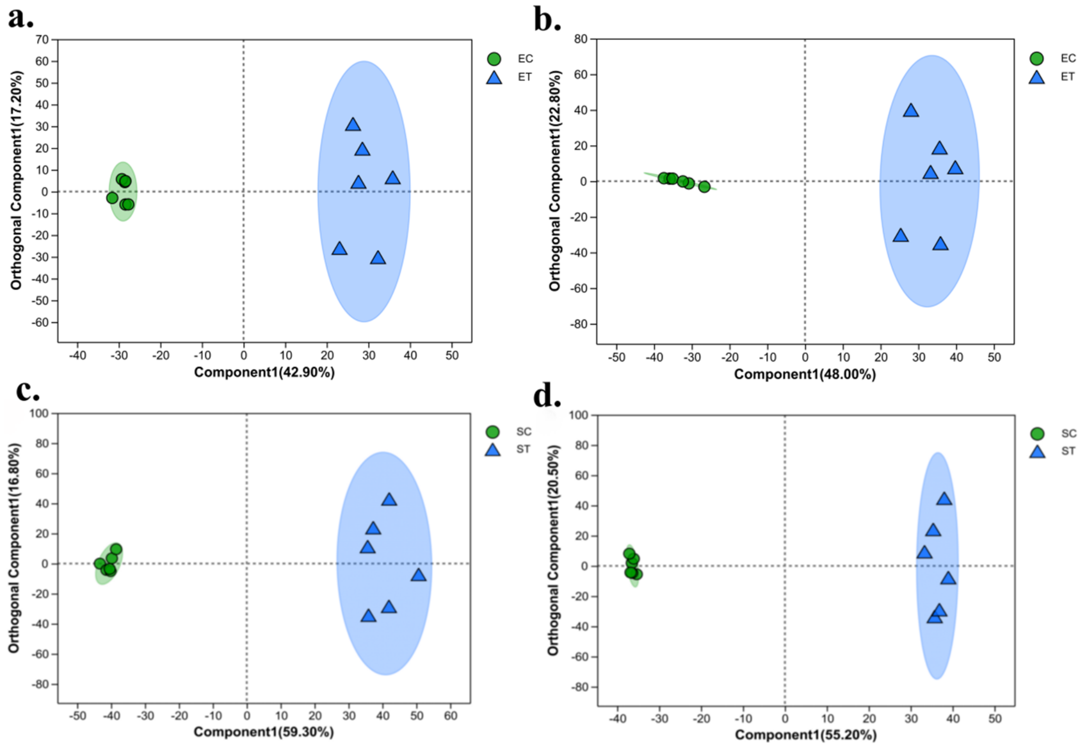

Figure S6 Metabolomics response. *E. coli* positive (a) and negative (b) OPLS-DA score plot. *S. enteritidis* positive (c) and negative (d) OPLS-DA score plot.

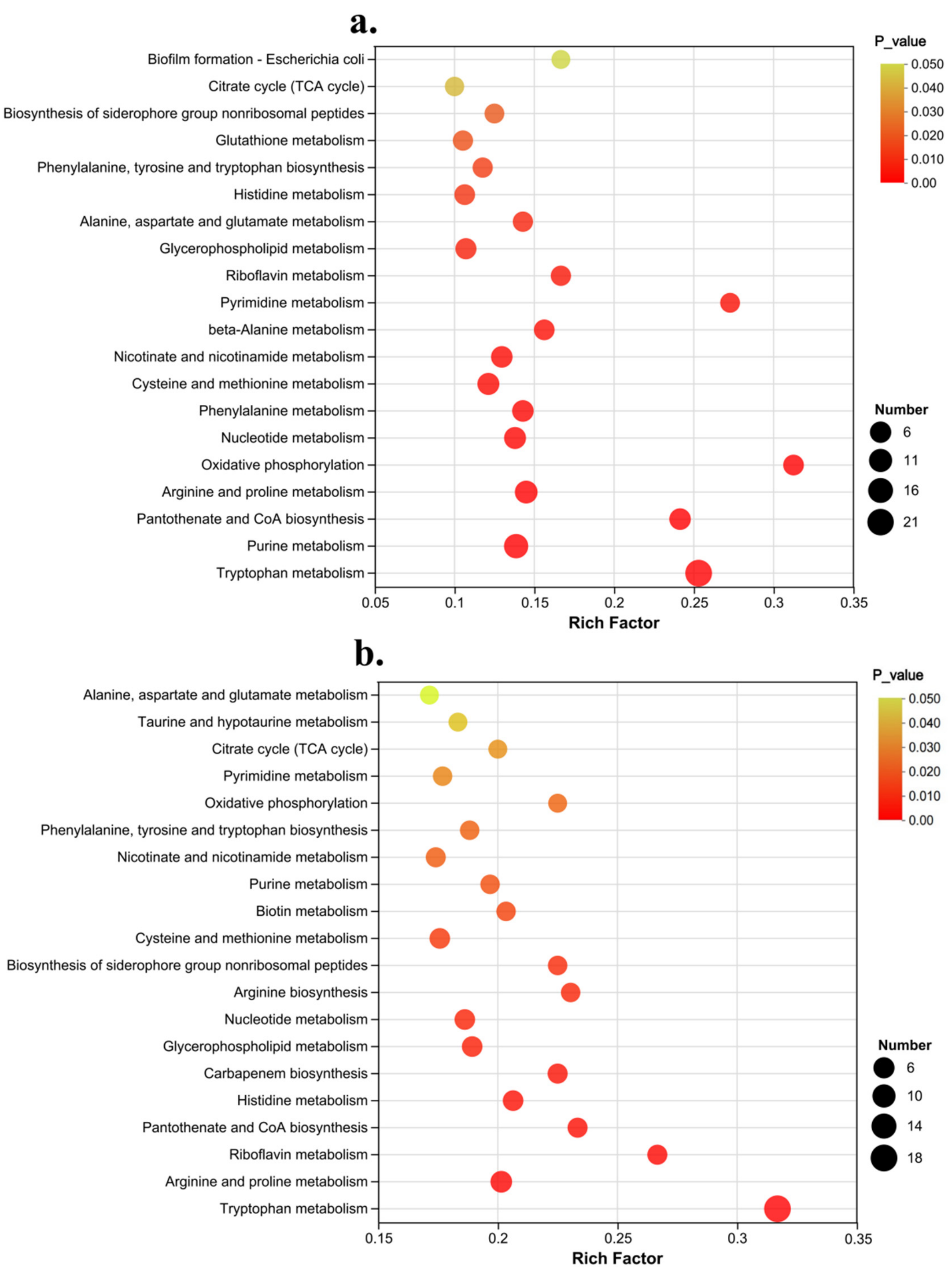

Figure S7 Metabolomics response. KEGG-enriched scatter plot of differential metabolites in *E. coli* (a) and *S. enteritidis* (b) after treatment with CPF-Fe.
